# From crystal to structure with CCP4

**DOI:** 10.1107/S0907444911007578

**Published:** 2011-03-18

**Authors:** Kevin Cowtan, Paul Emsley, Keith S. Wilson

**Affiliations:** a Structural Biology Laboratory, Department of Chemistry, University of York, Heslington, York, YO10 5DD, United Kingdom; b Department of Biochemistry, University of Oxford, South Parks Road, Oxford OX1 3QU

**Keywords:** CCP4

## Abstract

An introduction to the proceedings of the CCP4 study weekend is given.

The CCP4 Study Weekend 2010 was held at the University of Nottingham. Macromolecular crystallography is increasingly being performed by scientists who do not regard themselves as dedicated crystallographers, with more than half of the structures deposited in the Protein Data Bank having a first author who is submitting for the first or second time. Regular attendants to the CCP4 Study Weekend know that the audience has become larger and is composed of scientists with wider biological interests who use crystallography as but one of a number of tools. With this in mind, the 2010 Study Weekend was organised to provide an overview of macromolecular crystallography techniques with an emphasis on software and the *CCP*4 Suite in particular. Emphasis was placed on teaching students rather than fundamental research in software and tech­niques.

The *CCP*4 Suite has been at the forefront of macromolecular software for the last 30 years. The meeting was opened with a history of the Suite by one of its founders, Eleanor Dodson, followed by Martyn Winn who gave an overview of its current state and on-going developments. Zygmunt Derewenda then reviewed the current best practices in protein crystallization. This was followed by a presentation on the PiMS software by Chris Morris, replacing Rob Esnouf who was prevented from reaching the meeting due to the severe weather conditions.

The next session focussed on data acquisition, and the first speaker, Gwyndaf Evans, introduced the problems faced in designing the best experiment. In a highly relevant session for inexpert users, Andrew Leslie spoke about data integration and initial processing with *iMOSFLM*. Phil Evans introduced data reduction and the information on data quality it can provide, and Clemens Vonrhein gave valuable advice on handling routine and problematic cases.

The third session covered molecular replacement (MR). Gabor Bunkoczi spoke about automation of MR for complex cases in the *Phaser* software. Ronan Keegan described the database-driven automation of MR in the *MrBump* and *Balbes* pipelines. Bert Janssen gave a case study for a hard problem beyond current automation approaches. Gerard Kleywegt closed the session with an overview of changes at the PDBe.

The fourth session dealt with experimental phasing, with Raj Pannu talking about automation of experimental phasing from various sources in the *CRANK* pipeline. Randy Read talked about recent experience of SAD phasing in *Phaser*, and Pavol Skubak described some new developments in density modification to reduce the problems of bias in this method.

The fifth session covered refinement and model building. Dale Tronrud gave an overview of the different types of maps used during structure solution. Garib Murshudov introduced the latest features of the *Refmac* refinement software. Henry van den Bedem described the experience of the JCSG with different model building software in automated pipelines. Jane Richardson gave a guide on the use of *MolProbity* in the validation of macromolecular structures.

The final session included an introduction to the identification of protein complexes by Eugene Krissinel, and talks on *CCP4mg* by Stuart McNicholas and *Coot* visualization and validation software by Paul Emsley.

It now seems clear that continuing developments in integration and automation of the powerful software here described will soon lead to routine generation of high-quality (but perhaps not at the moment ‘fully polished’) novel structures from molecular replacement or experimental phasing generated within an hour of starting the data collection while at the synchrotron.

Some of the presentations provided excellent teaching material but were less suitable for publication in this issue. The reader is encouraged to view the available presentations online at the CCP4 website: http://www.ccp4.ac.uk/courses/stwk10/talks.html.

We thank the speakers for their contribution to the Study Weekend and the proceedings articles. We thank Shirley Miller, Damian Jones and Laura Johnston for organisational help and support.

